# Depression among Migrant and Left-Behind Children in China in Relation to the Quality of Parent-Child and Teacher-Child Relationships

**DOI:** 10.1371/journal.pone.0145606

**Published:** 2015-12-31

**Authors:** Jing Guo, Xuezhu Ren, Xiaohua Wang, Zhiyong Qu, Qianyun Zhou, Chun Ran, Xia Wang, Juan Hu

**Affiliations:** 1 Department of Sociology, Huazhong University of Science and Technology, Wuhan, P. R. China; 2 School of Education, Huazhong University of Science and Technology, Wuhan, P. R. China; 3 School of Social Development and Public Policy, Beijing Normal University, Beijing, P. R. China; 4 Beijing Haidian Institute for Educational Research, Beijing, P. R. China; 5 Community health service center, Huazhong University of Science and Technology, Wuhan, P. R. China; TNO, NETHERLANDS

## Abstract

The objective of this study was to examine rates of depression among migrant children (MC) and left-behind children (LBC) as compared to non-left-behind children (NLBC) and also to examine the relationship between depression among these children and the quality of their parent-child and teacher-child relationships. This study collected data from a large sample of 3,759 children aged from 8 to 17 years, including 824 who had been left behind by one parent (LBCO), 423 who had been left behind by both parents (LBCB), 568 MC and 1944 NLBC. Children’s Depression Inventory–Short Form was used to measure child depression. Parent-Child Relationship Scale (PCRS) and Teacher-Child Relationship Scale (TCRS) were used to measure the quality of parent-child and teacher-child relationships, respectively. The results showed that the prevalence of depression was 10.5% among NLBC, 13.1% among LBCO, 16.1% among LBCB, and 20.1% among MC. Depression was related to parent-child relationship quality and teacher-child relationship quality. Negative parent-child relationship was more relevant to depression than negative teacher-child relationship among LBCB, while negative teacher-child relationship was the most correlated with depression among MC.

## Introduction

In China, left-behind children (LBC) are defined as “children under 18 who have been left behind at their original residence while one or both parents migrate into other places for work, and have been not living together with them for at least six months”[1]. Migrant children (MC) are defined as “children under 18 who have left their original residence and migrated to a big city at least six months ago”[2]. Non-left-behind rural children (NLBC) are “children under 18 who live with both parents at their original residence”. With the rapid increase, in recent years, in the number of migrant workers who have moved from rural areas to cities or factory enclaves for better work opportunities, the numbers of MC and LBC have been growing fast in China. It is estimated that around 61 million children were left behind in their rural hometowns by their parents, and nearly 35.81 million migrant children relocated to cities with their parents, in 2013[3]. Numerous studies have found that MC and LBC are more likely to report poor interpersonal relationship satisfaction, and depression [4,5,6]. Therefore, the current study focused on depression among migrant and left-behind children and on the relationship between child depression and the quality of teacher-child and parent-child relationships.

In China, the majorities of migrant workers are employed in low-paying jobs and live in crowded conditions in cities. Moreover, China’s place-based public resource distribution and management systems pose formidable obstacles, for rural-to-urban migrants, to access to public goods like primary education and medical care [7]. Thus, a considerable number of migrant workers cannot afford to bring their children to the destination cities. A consequence of this is a long-term parental separation from children, which can be quite injurious to the mental health of the left-behind children. On the other hand, for those rural children who live with their parents in cities, they are not entitled to the same privileges as urban children because of the Hukou system of household registration. Consequently, most rural children are unable to enroll in city public schools. Although there are unregistered schools in cities set up specifically for migrant children, those schools are usually small, lack qualified teachers, and do not have standard teaching materials or adequate sanitation facilities[4]. The evidence that is available suggests that children of migrant workers may not be experiencing positive peer and teacher–student relationships [6]. Negative relationships with parents and teachers may, to a large extent, increase children's internalizing and externalizing problems.

Attachment theory provides a conceptual framework for understanding the impact of negative parent-child and teacher-child relationships on the likelihood in depression for MC and LBC. Early experiences with parent and teachers are important in developing secure attachment relationships. Children with secure attachment are more likely to o exhibit a more positive, integrated view of self, are more prone to positive self-disclosure, and cope more adaptively with stressful situations[8]. Experience of separation and confliction with their parents and teacher develop insecure attachment relationships for LBC and MC. Children with insecure attachment use less effective strategies in stressful situations and show higher frequencies of internalizing problems (e.g., depression). Both theoretical accounts and empirical work indicate that left-behind and migrant children are subject to mental health problems such as depression[5]. Recent studies suggest that MC and LBC are at increased risk for mental health problems, particularly depression and anxiety [9,10]. Compared to the general children population, LBC exhibit more psychological problems, such as low self-esteem, lack of self-confidence, loneliness, depression, emotional instability and social anxiety[11].Similarly, relatively high levels of separation anxiety, generalized anxiety disorder and depression have been found among MC [6]. Unfortunately, no prior study has simultaneously examined social and psychological risk factors associated with depression among MC and LBC as compared to rural children who live with their parents in their hometowns.

Previous research on children’s mental health, although not particularly focused on MC and LBC, has suggested that depression, internalizing and externalizing problems among children are closely related to the quality of their relationships with their parents[12], and with their teachers. These findings seem reasonable given the generally accepted framework of child development in which parent-child relationships and teacher-child relationships represent two critical factors influencing children’s mental health and behavior problems[13]. Specifically, families of children with depression tend to have persistently unstable and ineffective parent–child interactions [14], while children who perceive their relationships with their parents as satisfactory are less likely to be depressed [15]. Children’s relationships with their teachers are also important for their mental health given that school-aged children spend most of their weekdays in classrooms where they interact with teachers, who are the only available adults. Carlo Schuengel argues that studying children’s relationships with important adults in their lives other than their parents, such as teachers, is indispensable for a complete understanding of children’s socio-emotional development[16]. A number of studies have indicated that positive teacher–child relationships help protect children against depression [17,18].

An attachment perspective also guides research hypotheses regarding the consequences of adult–child relationship quality and the intervening mechanisms explaining these effects[16]. Investigations of the impact of interpersonal relationships on childhood depression are complicated by the fact that the effects on depression of teacher-child relationships and parent-child relationships may be interactive. A recent review suggests that children’s relationships with teachers and their parents may be concordant, but in some cases teacher-child relationships may moderate the effects of detached parent-child relationships on child risk [16]. Therefore, high-quality teacher–child relationships may protect or promote functioning among at-risk children. Therefore, it is important that studies of MC and LBC, who are considered to be at risk because of either changing living environments or family separation, take such moderating effects into consideration.

The aim of the present study is to extend existing research on depression among MC and LBC, by addressing both parent-child and teacher-child relationships. Using cross-sectional data on a large sample of Chinese children, we firstly examined whether MC, LBC, and NLBC differed with regard to depression. Secondly, we examined the compare effects of teacher-child relationship quality and parent-child relationship quality on children’s depression status across MC, LBC and NLBC.

## Methods

### Participants

The sample is comprised of 3,759 children aged between 8 to 17 years, including 824 LBCO, 423 LBCB, 568 MC, and 1944 NLBC. The LBC and NLBC were recruited from nine primary and middle schools in three rural counties of Henan Province and Shanxi Province. The sample of MC was randomly selected from three schools for migrant children located in Beijing. Over the past decade, Henan and Shanxi have been the major labor-exporting provinces, and Beijing has been the major labor-importing city in China.

Given the high mobility and the unregistered status of migrants in China, it is difficult to obtain a random sample of migrants for a study [19]. The sampling procedure of the present study was as follows: (1) educational statistics for the selected area were examined, and primary and middle school principals were interviewed, to determine what proportion of the children in the rural schools of selected area were left-behind children; (2) schools sharing similar characteristics in terms of social recognition and financial appropriation from local governments were selected. In each of the selected schools, one class was randomly selected from each grade (4th-9th). The eligibility criteria for LBC participants were: (1) they had been born and raised in the countryside; and (2) one or both of their parents had migrated to a city for employment. The selected LBC were divided into two groups, LBCO and LBCB, according to whether they had been left behind by one or both parents. Children still living with both parents (NLBC) were recruited from the same classes as the left-behind children. Aside from the 2.0% of the selected students who declined to participate in the study, the other selected students agreed to participate and provided informed consent. The participants were asked to complete a self-administered questionnaire, if they were from the higher grades. For younger students from the lower grades, trained interviewers (including one university teacher and 19 graduate students in psychology) provided assistance by explaining survey items to them.

This study was approved by the Human Subjects Review Committee of the School of Social Development and Public Policy at Beijing Normal University. Approved verbal informed consent was used rather than signatures or fingerprints, because the latter were regarded as sensitive issues in the local culture. We obtained informed consent from next of kin, caretakers, or guardians on behalf of the children enrolled in our study. Verbal consent was also obtained from the children after they were informed the aim of the survey and their rights to refuse to participate.

### Measurement

1. **Demographic information** A number of demographic and economic variables were collected, including gender (male, female), age category (under 11, 11–13, 14 and above), parental education attainment (primary school or below, junior high school, senior high school or above), and self-reported family financial situation (poor, middle class, rich).
2. **Depressive symptoms** were assessed using the Children’s Depression Inventory-Short Form (CDI-S). The full Children’s Depression Inventory has been widely used to diagnose depression in children according to the *Diagnostic and Statistical Manual of Mental Disorders* (4th edition) criteria. CDI-S has been found to have a correlation of 0.89 with the full inventory[20]. It is the most commonly used valid and reliable screening tool for depressive symptoms in 7- to 17-year olds in China[10]. This questionnaire was composed of 10 items. For each item children were asked to choose which of three statements best described their feelings in the past 2 weeks. Responses to each item were then rated 0 to 2, with 0 being assigned for the statement indicating the least depressed mood and 2 being assigned to the statements endorsing the most depressed mood. The total score was calculated by adding the scores of all the items (range, 0–20). Depression was defined as a CDI-S total score greater than or equal to 7 based on international norms [21,22]. The internal consistency coefficient for the CDI-S for this sample was Cronbach’s α = 0.75.
3. **The Teacher–Child Relationship Scale (TCRS) **is a 28-item rating scale, using a Likert-type format, designed to assess students’ perceptions of their relationship with a particular teacher. It was revised from Pianta’s Student–Teacher Relationship Scale (STRS) [23]. Response categories were “Almost Never or Never”, “Seldom”, “Sometimes”, “Often”, and “Almost Always or Always”. The two extreme responses were scored as 1 or 5, depending on whether an item was positively or negatively worded. The Chinese version of the TCRS has been demonstrated to have satisfactory psychometric properties, comparable to those of the original English version[24]. Exploratory factor analysis has indicated that the STRS items are best represented by four factors representing conflict, closeness, supportiveness and satisfaction[24]. We conduct confirmatory factor analysis to validate the factor structure. The STRS measures student perceptions on these relationship dimensions: the closeness subscale reflects the degree of openness, warmth, and security in the relationship as perceived by the student; the conflict subscale measures the degree to which a student perceives teacher–student interactions as negative, discordant, unpredictable, and unpleasant; the supportiveness subscale measures the extent to which the child feels supported by the teacher; and the satisfaction subscale measures the extent to which the student cherishes their relationship with the teacher. Higher scores on the closeness, supportiveness, and satisfaction subscales and lower scores on the conflict subscale indicate more positive teacher–child relationships. The internal consistency coefficient assessed by Cronbach’s α for the teacher-child relationship scale in this sample was 0.93. The internal consistency coefficient assessed by Cronbach’s α for conflict, closeness, supportiveness and satisfaction in this sample was 0.86, 0.82, 0.79, and 0.76.
4. **The original version of the Parent–Child Relationship Scale (PCRS) was used in** the development of the Inventory of Parent and Peer Attachment (IPPA), a self-report instrument for use with children[25]. The Chinese version of the PCRS has been found to have satisfactory psychometric properties [26]. Subjects complete a 15-item questionnaire by indicating how often each statement is true for them on a 5-point Likert scale. Response categories are “Almost Never or Never”, “Seldom”, “Sometimes”, “Often”, and “Almost Always or Always”. The items were demonstrated through principal components analysis to cluster into three factors (trust; communication; anger and alienation) [26]. The items were designed to assess the child's trust that her/his mother understands and respects her/his needs and desires, and is sensitive and responsive to her/his emotional states and concerns (security). Items assessing anger toward or emotional detachment from the mother are also included, since frequent and intense anger or detachment is seen as a response to actual or threatened disruption of the mother-child bond. Higher trust scores, higher communication scores, and lower alienation scores indicate more positive relationships. The internal consistency indexed by Cronbach’s α for the parent-child relationship scale in this sample was 0.88. The internal consistency coefficient assessed by Cronbach’s α for trust, communication, and alienation in this sample was 0.78, 0.81, and 0.53.

### Statistical analysis

Our preliminary analyses produced descriptive statistics for the independent and dependent variables. Chi-square analyses were conducted to test for between-group differences in the depression and demographic variables. ANOVA and Post hoc analysis was used to examine the relationships between depression scores and the individual dimension scores of the interpersonal relationship scales, among MC, LBC and NLBC. It is hard to run a simple regression with interaction terms since the PCRS contains three subscales, and the TCRS contains four subscales, which reflecting different richness information. Cluster analysis of the teacher-child and parent-child relationship quality data was used to reduce complexity by generating groups with shared characteristics. The data was break down into two clear groups: positive interpersonal relationship and negative interpersonal relationship. The primary analyses consisted of a series of regression analyses for depression. Logistic regression analysis was also used to examine the relationship among type of child (MC, LBC or NLBC), quality of interpersonal relationships, and depression. Additional logistic regression analyses were applied to see what specific aspects of the relationships are most predictive of depression. Then, the whole sample was divided into 4 groups according to quality of interpersonal relationships to examine the compare effect of parent-child relationship quality (Positive/negative), and teacher-child relationship quality (Positive/negative) on depression. Multiple logistic regressions were employed to examine the effect of parent-child relationship quality and teacher-child relationship quality on depression among MC, LBC, and NLBC respectively.

The analyses were conducted using the SPSS statistical software program (PASW 18.0).

## Results

### Descriptive results

The rates of depression and of demographic characteristics for each child group are displayed in Table 1. The sample consisted of children aged between 8 and 17 years old, with a mean age of 12.64 (Standard Deviation, SD = 1.91). There was an almost even gender distribution among the LBC and NLBC, but there were more boys than girls among the MC (57.0% vs. 43.0%). The prevalence of depression in the overall sample was 13.0%, but rates of depression were higher for the MC (20.1%), LBCO (13.1%), and LBCB (16.1%), and lower for the NLBC(10.5%). Post-hoc comparison showed that MC had higher rates of depression than LBCO and NLBC. Compare to LBC and MC, NLBC had a lower rates of depression.

**Table 1:** Depression rates and demographic characteristics of NLBCs, LBC and MC(N = 3759).

	NLBC (*n* = 1944)	LBC(*n* = 1247)		MC(n = 568)		
		*LBCO (824)*	*LBCB (423)*			
	*n* (*%*)	*n* (*%*)	*n* (*%*)	*n* (*%*)	χ2	P-value
**Depression**						<0.001^c^
No	1740(89.5)	716(86.9)	355(83.9)	454(79.9)		
Yes	204(10.5)	108(13.1)	68 (16.1)	114(20.1)	39.02	
**Demographics**						
Gender						0.041
male	979(50.4)	419(50.8)	214(50.6)	324(57.0)		
female	965(49.6)	405(49.2)	209(49.4)	244(43.0)	8.28	
Age group						<0.001
under 11	400(20.6)	120(14.6)	76(18.0)	239(42.1)		
11–13	932(47.9)	456(55.3)	220(52.0)	281(49.5)		
14 and above	612(31.5)	248(30.1)	127(30.0)	48(8.5)	224.07	
Mother’s education level^a^						<0.001
Primary school/ below	599(31.0)	353(42.9)	123(29.2)	212(37.7)		
Junior high school	900(46.5)	335(40.8)	214(50.8)	227(40.4)		
Senior high school/above	435(22.5)	134(16.3)	84(20.0)	123(21.9)	50.42	
Family financial situation^b^						<0.001
Rich	147(7.6)	41(5.0)	32(7.7)	109(19.6)		
Common	1303(67.8)	511(62.4)	249(60.0)	378(68.0)		
Poor	472(24.6)	267(32.6)	134(32.3)	69(12.4)	157.08	

Note: NLBC = Non-left-behind rural children, LBC = Children who were left behind, LBCO = Children who were left behind by one parent, LBCB = Children who were left behind by both parents, MC = Migrant children. a&b have some missing data.

^a.^ N = 3739;

^b.^ N = 3712.

^c.^ Post-hoc comparison showed that MC had higher rates of depression than LBCO and NLBC.

Compare to LBC and MC, NLBC had a lower rates of depression.

In order to examine the relationship between depression and interpersonal relationship quality among NLBC, LBC and MC, ANOVA and post-hoc analysis were conducted. The results are summarized in Table 2. There were higher scores of depression for the MC, and LBCB than for the other groups. There were significant differences between different groups of children with respect to trust(F_(3,3755)_ = 50.50, p<0.001), communication(F_(3,3755)_ = 46.35, p<0.001), conflict(F_(3,3755)_ = 8.68, p<0.001), supportiveness(F_(3,3755)_ = 10.41, p<0.001), and satisfaction(F_(3,3755)_ = 6.03, p<0.001). Compared to the other groups, the MC had the lowest scores on the trust and communication dimension of parent-child relationship quality, and had the highest scores on the alienation dimension. With respect to the teacher-child relationship quality, MC had the lowest scores on the supportiveness and satisfaction dimensions, and the highest scores on the conflict dimension.

**Table 2:** Comparisons among NLBC, LBC and MC regarding depression and quality of interpersonal relationships (N = 3759).

	NLBC(*n* = 1944)	LBC(*n* = 1247)		MC(n = 568)		
		*LBCO (824)*	*LBCB (423)*			
	*Mean(SD)*	*Mean(SD)*	*Mean(SD)*	*Mean(SD)*	F^a^	PC^b^
**Depression**	3.12(2.90)	3.41(2.80)	3.71(3.05)	4.06(2.90)	19.11***	MC > NLBC, LBCO***, LBCB>NLBC***
**Parent-child relationship**						
Trust	3.74(0.83)	3.70(0.81)	3.79(0.87)	3.27(0.94)	50.50***	MC < NLBC, LBC***
Communication	3.26(0.98)	3.31(0.91)	3.41(0.98)	2.79(1.05)	46.35***	MC < NLBC, LBC***
Alienation	2.27(0.70)	2.26(0.65)	2.23(0.67)	2.31(0.69)	1.27	
**Teacher-child relationship**						
Closeness	3.21(0.94)	3.26(0.91)	3.25(0.91)	3.15(0.91)	1.79	
Conflict	1.94(0.81)	1.86(0.80)	1.95(0.80)	2.11(0.89)	8.68***	MC > NLBC, LBC ***
Supportiveness	3.35(0.52)	3.43(0.54)	3.34(0.55)	3.24(0.63)	10.41***	MC < NLBC, LBC ***
Satisfaction	3.75(0.89)	3.34(0.55)	3.76(0.89)	3.61(0.95)	6.03***	MC < NLBC, LBCB ***

Note: NLBC = Non-left-behind rural children, LBC = Children who were left behind, LBCO = Children who were left behind by one parent, LBCB = Children who were left behind by both parents, MC = Migrant children.

*** = p<0.001.

^a.^ The F value was from ANOVA results.

^b.^ PC indicate the significance of pairwise comparisons in the post-hot analysis.

LBCB> NLBC *** means “LBCB had a significant higher score than NLBC”.

MC> NLBC, LBC *** means “MC had a significant high score compared with NLBC, LBCO and LBCB”.

MC< NLBC, LBC *** means “MC had a significant low score compared with NLBC, LBCO and LBCB”.

MC< NLBC, LBCB *** means “MC had a significant low score compared with NLBC and LBCB”.

Cluster analyses of Q-Set items were used to categorize the parent-child and teacher-child relationships as positive or negative. The data was break down into two clear groups. “Positive parent-child relationship” and “Negative parent-child relationship” were abbreviated as PPCR and NPCR respectively. “Positive teacher-child relationship” and “Negative teacher-child relationship” were abbreviated as PTCR and NTCR. Independent-Sample T Tests were performed to externally validate these categories. The results of the independent-sample test are presented in Table 3. The positive parent-child relationship group (n = 2289; 60.9%) consisted of high trust and communication scores, and those with negative parent-child relationships (n = 1470; 39.1%) were more likely to have high alienation scores. The positive teacher-child relationship group (n = 1921; 51.1%) consisted of high closeness, supportiveness and satisfaction scores, and those with negative teacher-child relationships (n = 1838; 48.9%) were more likely to have high conflict scores.

**Table 3:** Parent-child relationship and teacher-child relationship quality ratings (Positive/Negative) by subscale scores(N = 3759).

	Parent-child relationship						Teacher-child relationship							
	Trust		Communication		Alienation		Closeness		Conflict		Supportiveness		Satisfaction	
	Mean	SD	Mean	SD	Mean	SD	Mean	SD	Mean	SD	Mean	SD	Mean	SD
Positive	4.17	0.51	3.83	0.62	2.14	0.64	3.82	0.70	1.48	0.57	3.62	0.40	4.39	0.52
Negative	2.88	0.69	2.26	0.64	2.47	0.70	2.57	0.70	2.46	0.74	3.06	0.54	3.06	0.70
T-test	65.63***		73.75***		-14.88***		54.84***		-45.70***		36.38***		66.55***	

Note:

*** = p<0.001.

### Regression results

Table 4 presents the logistic regression analysis results for depression by type of child, interpersonal relationship quality ratings, and demographic characteristics. Two models were investigated in this analysis. The seven dimension variables of the interpersonal relationship quality scales were included in Model 1. The two dichotomous variables of the interpersonal relationship quality scales were included in Model 2. The results show that, adjusting for demographic information, child type, parent-child relationship quality, and teacher-child relationship quality are related to depression. Model 1 indicated that the degree of trust(OR, 0.61; 95% CI, 0.51–0.72), communication(OR, 0.82; 95% CI, 0.71–0.96), and alienation(OR, 1.75; 95% CI, 1.50–2.03) of the parent-child relationship were significantly related to depression. In addition, children who have conflict relationship with their teacher reported more depression (OR, 1.54; 95% CI, 1.34–1.76), and those who have satisfaction relationship with their teacher reported less depression (OR, 0.81; 95% CI, 0.68–0.96).

**Table 4:** Regression analysis for depression by type of child, interpersonal relationship quality ratings, subscale scores, and demographic characteristics (N = 3696).

	*Model 1*			*Model 2*		
	*OR*	*95%*	*P*	*OR*	*95%*	*P*
**Types of children NLBC = ref.**						
LBCO	1.22	0.93–1.60	0.154	1.18	0.91–1.53	0.221
LBCB	1.93	1.38–2.68	<0.001	1.87	1.36–2.57	<0.001
MC	1.56	1.16–2.10	0.003	1.70	1.28–2.25	<0.001
**Parent-child relationship**						
trust	0.61	0.51–0.72	<0.001			
communication	0.82	0.71–0.96	0.014			
alienation	1.75	1.50–2.03	<0.001			
**Teacher-child relationship**						
closeness	1.24	1.05–1.47	0.014			
conflict	1.54	1.34–1.76	<0.001			
supportiveness	0.79	0.63–1.00	0.050			
satisfaction	0.81	0.68–0.96	0.015			
**Parent-child relationship** PPCR = ref.						
NPCR				2.94	2.37–3.64	<0.001
**Teacher-child relationship** PTCR = ref.						
NTCR				2.18	1.74–2.71	<0.001
**Gender** male = ref.						
female	1.05	0.85–1.29	0.686	1.03	0.84–1.26	0.777
**Age group** above 14 = ref.						
under 11	1.41	1.02–1.94	0.037	1.26	0.93–1.71	0.132
11–14	1.03	0.79–1.33	0.845	1.01	0.79–1.30	0.918
**Mother’s education level** Senior high school or above = ref.						
Primary school or less	1.15	0.85–1.54	0.363	1.19	0.90–1.58	0.228
Junior high school	0.86	0.64–1.15	0.298	0.83	0.63–1.10	0.200
**Perceived family financial situation**Rich = ref.						
Middle class	1.04	0.70–1.54	0.845	0.94	0.65–1.38	0.763
Poor	2.09	1.38–3.15	<0.001	2.02	1.36–3.01	0.001

**Note:** NLBC = Non-left-behind rural children, LBC = Children who were left behind, LBCO = Children who were left behind by one parent, LBCB = Children who were left behind by both parents, MC = Migrant children. PPCR = Positive parent-child relationship, NPCR = Negative parent-child relationship, PTCR = Positive teacher-child relationship, NTCR = Negative teacher-child relationship

In further regression analysis, there was significant interaction between closeness and conflict. Due to limited space, this will not delve into the details. The Model 2 results show that, compared to the NLBC, LBCB were more likely to be depressed (OR, 1.87; 95% CI, 1.36–2.57), as were MC (OR, 1.70; 95% CI, 1.28–2.25). Negative parent-child relationship quality almost tripled the risk of depression (OR, 2.94; 95% CI, 2.37–3.64). Children with perceived negative teacher-child relationships were twice as likely to be depressed as those with positive teacher-child relationships (OR, 2.18; 95% CI, 1.74–2.71).

Table 5 presents the regression analysis results for depression by interpersonal relationship quality among NLBC, LBC and MC. Four models were investigated to examine the effect of parent-child relationship quality and teacher-child relationship quality on depression among MC, LBCO, LBCB, and NLBC respectively. The results show that, adjusting for demographic information, parent-child relationship quality interacts with teacher-child relationship quality to significantly affect the risk of depression for MC, LBC, and NLBC respectively (Models 1–4). The Model 1 results show that children with perceived negative parent-child relationships and positive teacher-child relationships are nearly five times as likely to be depressed as those for whom both relationships are positive (OR, 4.58; 95% CI, 2.66–7.88) among NLCB. Children with perceived positive parent-child relationships and negative teacher-child relationships are nearly three times as likely to be depressed as those for whom both relationships are positive (OR, 2.59; 95% CI, 1.55–4.33). The Model 2 results show that children with perceived negative parent-child relationships and positive teacher-child relationships are nearly three times as likely to be depressed as those for whom both relationships are positive (OR, 2.84; 95% CI, 1.33–6.07) among LCBO. Children with perceived positive parent-child relationships and negative teacher-child relationships are nearly three times as likely to be depressed as those for whom both relationships are positive (OR, 2.42; 95% CI, 1.23–4.77). The Model 3 results show that children with perceived negative parent-child relationships and positive teacher-child relationships are nearly five times as likely to be depressed as those for whom both relationships are positive (OR, 4.67; 95% CI, 1.93–11.31) among LBCB, while the effect of negative teacher-child relationship is non-significant for those with positive parent-child relationships. The Model 4 results show that migrant children with negative teacher-child relationships and positive parent-child relationships are significantly more likely to be depressed than those for whom both relationships are positive (OR, 3.01; 95% CI, 1.29–6.99), while the effect of negative parent-child relationship is non-significant for MC with positive teacher-child relationships. In addition, negative parent-child and negative teacher-child relationships have significant negative impact on all groups.

**Table 5:** Regression analysis for depression by interpersonal relationship quality among NLBC, LBC and MC.

	*Model 1-NLBC* ^a^			*Model 2-LBCO* ^b^			*Model 3-LBCB* ^c^			*Model 4-MC* ^d^		
	*OR*	*95% CI*	*P-value*	*OR*	*95% CI*	*P-value*	*OR*	*95% CI*	*P-value*	*OR*	*95%CI*	*P-value*
**Quality of relationships** PPCR&PTCR = ref.												
NPCR&PTCR	**4.58**	**2.66–7.88**	**<0.001**	**2.84**	**1.33–6.07**	**0.007**	**4.67**	**1.93–11.31**	**0.001**	1.98	0.85–4.66	0.116
PPCR&NTCR	**2.59**	**1.55–4.33**	**<0.001**	**2.42**	**1.23–4.77**	**0.011**	2.59	0.84–4.31	0.122	**3.01**	**1.29–6.99**	**0.010**
NPCR&NTCR	**7.59**	**4.77–12.08**	**<0.001**	**6.73**	**3.68–12.32**	**<0.001**	**6.33**	**3.04–13.18**	**<0.001**	**5.70**	**2.74–11.85**	**<0.001**
**Gender** male = ref.												
female	0.92	0.68–1.25	0.603	1.17	0.76–1.80	0.480	0.68	0.38–1.21	0.189	1.42	0.91–2.22	0.127
**Age group** above 14 = ref.												
under 11	1.21	0.78–1.88	0.388	1.11	0.55–2.25	0.771	1.35	0.54–3.37	0.524	1.73	0.70–4.25	0.236
11–14	0.88	0.62–1.25	0.469	1.00	0.61–1.64	0.988	1.68	0.86–3.29	0.129	1.25	0.52–3.04	0.622
**Mother’s education level** Senior high school/above = ref.												
Primary/below	1.46	0.93–2.28	0.100	1.29	0.68–2.46	0.434	1.59	0.69–3.65	0.277	0.61	0.34–1.07	0.084
Junior high school	0.98	0.63–1.52	0.923	0.72	0.36–1.43	0.345	0.96	0.43–2.12	0.912	0.64	0.36–1.11	0.113
**Family financial situation** Rich = ref.												
Common	1.18	0.59–2.38	0.646	0.57	0.21–1.51	0.256	0.69	0.23–2.11	0.514	1.10	0.62–1.96	0.753
Poor	2.34	1.13–4.81	0.021	1.39	0.53–3.70	0.505	1.56	0.51–4.79	0.440	2.41	1.15–5.07	0.020

Note, NLBC = Non-left-behind rural children, LBC = Children who were left behind, LBCO = Children who were left behind by one parent, LBCB = Children who were left behind by both parents, MC = Migrant children. PPCR = Positive parent-child relationship, NPCR = Negative parent-child relationship, PTCR = Positive teacher-child relationship, NTCR = Negative teacher-child relationship

a, b, c, d have missing data.

^a.^ N = 1912;

^b.^ N = 817;

^c.^ N = 413,

^d.^ N = 554.

**Fig 1:**
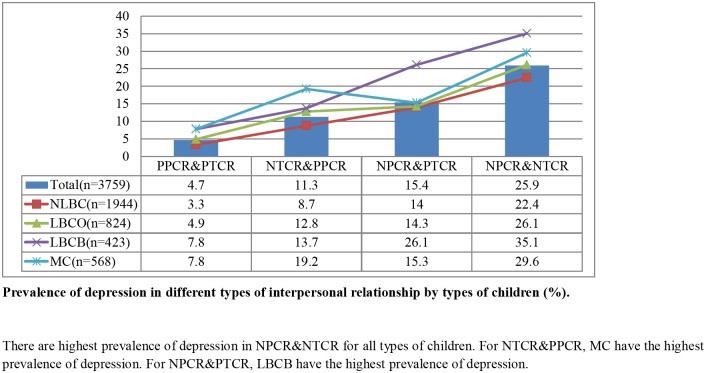
Prevalence of depression in different types of interpersonal relationship by types of children (%). There are highest prevalence of depression in NPCR&NTCR for all types of children. For NTCR&PPCR, MC have the highest prevalence of depression. For NPCR&PTCR, LBCB have the highest prevalence of depression.

Finally, the compare effect of parent-child relationship and teacher-student relationship quality was significantly related to depression. This pattern is illustrated in Fig 1. The rate of depression was as high as 26.1% for LBCB who had negative parent-child relationships and positive teacher-child relationships, compared to 13.7% for those with negative teacher-child relationships and positive parent-child relationships, suggesting that, among LBCB, depression is most strongly affected by the parent-child relationship. Among MC, the depression rate was a high 19.2% for those having negative teacher-child relationships and positive parent-child relationships, compared to 15.3% for those having negative parent-child relationships and positive teacher-child relationships, suggesting that, among MC, depression is most strongly affected by the teacher-child relationship (Fig 1).

## Discussion

The present study sought to examine the relationship among parent–child relationship quality, teacher–child relationship quality, and depression, among MC, LBCB, LBCO, and NLBC. The results indicate that MC and LBCB are at higher overall risk for depression than the other groups. MC are more likely to have negative parent-child and teacher-child relationships. In addition, negative parent-child relationship is more relevant to depression than negative teacher-child relationship among LBCB, while negative teacher-child relationship is the most correlated with depression among MC.

The results suggest that rates of depression are significantly higher among MC and LBCB than other groups. Migrant workers often faced a hard choice as parents: “Bring the child along or leave her/him behind?” The results of the present study indicate that the rate of depression among migrant children is as high as 20.1%, as compared to16.1% among children left behind by both parents. Prior studies have reported higher rates of depression and anxiety among LBC than among their age-matched peers[10]. This study contributes to the literature by comparing different groups of children affected by migration, and adds information concerning the relationship between migration and health. Particularly striking is the finding that MC are the most vulnerable group with regard to mental health. Migration entails not only loss of family and friends and the sense of home, but also the need to adapt to a new cultural environment that involves different sets of moral values and standards. These factors are likely to lead to a high level of stress and strain for MC. Poverty may also add to the stress for MC. Since many migrants originate from villages with low educational resources, and then have to start “from scratch” in their new homes, migrant families frequently suffer from poverty, unemployment and problems finding accommodation [27]. Indeed the present finding indicates that there was a significant relationship between perceived poverty and depression among MC children. Another factor contributing to depression may be the educational inequalities that adversely affect migrant children. In 2003, the General Office of the State Council of China issued a document advocating that migrant children be provided with equal access to education, and public schools be prohibited from charging them extra fees. However, this policy has yet to be implemented [4].

An important finding of the current study is that MC were significantly more likely to have negative/detached relationships with their parents and teachers than LBC and NLBC. Although MC live with their parents, they may receive insufficient care from their parents. The occupations of most migrant parents tend to be extremely time consuming, laborious, and unstable, thus interfering with their ability to spend time with their children [28]. Experiences of stigmatization might also affect parent-child relationships for MC. Prior study indicate that rural-to-urban migrant workers in China experience various forms of stigmatization including labelling, stereotyping, separation, status loss and discrimination[29]. In addition, the occupational and residential instability of parents (they tend to frequently change jobs and move from one worksite to another) leads to repeated changing of schools for MC. Not only would this affect the development of peer and student–teacher relationships among children of migrant workers, it may also further increase their sense of isolation and reduce their sense of belonging. Furthermore, there is evidence that urban public schools are generally well-resourced with qualified teachers, but in contrast the private migrant schools often have poor resources and conditions, with many under-qualified teachers. Teachers in public schools showed significantly more positive attitudes to inclusion of migrant students than migrant school teachers[30]. A report on 300 migrant schools in Beijing, for example, showed that only 63 were licensed. Teachers’ wages in those migrant schools were low and the workload was intense. Many teachers accepted jobs in migrant schools only as a stepping stone to a better position in public schools. As a result, the teacher turnover rate was high, disrupting the learning schedules of students [31]. Thus, it is not surprising that migrant students in these schools tend to perceive low intimacy and support, and high conflict, in their relationships with their teachers.

Another important finding is that children with negative parent-child and teacher-child relationships were more likely to be depressed. Negative parent-child and negative teacher-child relationships have significant negative impact on all groups. This is consisted with the view of attachment theory that adult-child relationship is vital for children mental health. Furthermore, the negative parent-child relationship has significant impact for NLBC, LBCO, and LBCB. The negative teacher-child relationship has significant impact for NLBC, LCBO and MC. Prior studies suggest that a positive teacher-student relationship can function as a “corrective experience”, and moderate the negative influences of a poor and conflictive parent-child relationship [32]. The current work indicates that negative parent-child relationship emerged as a more relevant factor related to depression than negative teacher-child relationship for LBCB, while negative teacher-child relationship was the most correlated with depression for MC. Because parental migration leads to the separation from parents for LBC, it may contribute to an unfavorable home environment for their social development. Thus negative parent-child relationship increased the likelihood of depression among LBC. The implication is that parents should try to maintain communication with LBC and support them, even if they cannot live with them. Teacher-child relationship quality is especially important for migrant children, perhaps because these children may got support and guidance from their teachers to partially compensate for the disadvantages with which their migrant status burdens them [33]. This finding suggests that interventions focused on improving teacher-child relationships may enhance the mental health of MC.

## Limitations and Implications

Several shortcomings of the present study should be pointed out. First, the current work cross-sectional in nature, and future longitudinal research is required to consolidate the current findings. Second, the use of a self-reported continuous measure of depression instead of a clinical diagnosis is another limitation of this study. Cautions should be taken to generalize the present findings to clinical condition. In addition, the cut-score is lack of external validity because these are depressive symptoms perceived by the children without any external diagnostic information to validate. Third, more variables related with families should be considered in future research. Many related factors (i.e., family social capital, the local cost of living and cost of housing, et al.) may influence a parent’s decision to either leave their children behind or migrate with them. Clarifying those factors is of assistance in further understanding the parent-child relationship. Moreover, children’s relationship with their caretakers may explain more variability in the children’s mental health than that of relationship with absent parents for the left behind children. It would also be informative to learn more about the amount of time that they are spending with adult caretakers for the migrant children.

The findings of the present work contributed significantly to the general mental health research literature and have important implications for the development of intervention and prevention services aimed at promoting child mental health. Given the significant correlation between the parent-child relationship, the teacher-child relationship quality and child mental health, strategies and programs should be developed to help children of migrant workers and their parents to develop better communication and problem-solving skills. Efforts must also be made to tackle the children’s educational disadvantages by developing programs that facilitate better understanding and respect between children of migrant workers and their teachers. At the community level, efforts should be made to build public facilities and create public spaces that can facilitate interaction between adults and children in neighborhoods, thus building up the stock of social capital. In addition, since left-behind children are vulnerable to emotional difficulties, special attention must be paid to these children to help them overcome the challenges induced by the absence of their parents. It is also of importance to help left-behind children maintain regular and positive communications with their migrant parents.

## Conclusions

The results show that MC and LBCB are at increased risk of depression. Negative parent-child relationship is more relevant to depression than negative teacher-child relationship among LBCB, while negative teacher-child relationship is the most correlated with depression among MC. Negative relationships with both parents and teachers were doing the worst on the depression regardless which group they were in. These results support the need for further research on negative interpersonal relationships as a risk factor for depression, and the need for programs aimed at improving the parent and teacher relationships of vulnerable children. Furthermore, the mental health needs of LBC may differ from those of MC.
